# Cell Invasion Analysis of Tumor Spheroids Using 2D Image Data

**DOI:** 10.1021/acsmeasuresciau.5c00121

**Published:** 2025-11-27

**Authors:** Matěj Přikryl, Andrea Rousová, Ivana Acimovic, Petr Vaňhara, Lukáš Jan, Petr Beneš, Jan Šmarda, Michal Kozubek, Karel Štěpka, Jarmila Navrátilová

**Affiliations:** 1 Department of Experimental Biology, Faculty of Science, 117204 Masaryk University, Brno 625 00, Czech Republic; 2 Department of Histology and Embryology, Faculty of Medicine, 117204 Masaryk University, Brno 625 00, Czech Republic; 3 Centre for Biomedical Image Analysis, Department of Visual Computing, Faculty of Informatics, 117204 Masaryk University, Brno 602 00, Czech Republic; 4 International Clinical Research Center, St. Anne′s University Hospital Brno, Brno 602 00, Czech Republic

**Keywords:** image analysis, software tool, object detection, 3D models, spheroids, fluorescence, invasion

## Abstract

Metastatic disease is the most severe complication in oncological patients. The quantification of cellular invasion into the surrounding tissue is crucial for the identification of strategies to suppress this process. Extracellular matrix-embedded 3D cancer models, such as spheroids and organoids, are commonly used to mimic tumor progression under *in vitro* conditions. However, robust and widely used algorithms to detect and quantify spheroid growth and invasion into the surrounding matrix are still lacking. In this study, we use fluorescently labeled 3D models, as fluorescence images are generally of higher quality than bright-field images. We present a methodology to compute the mask of the spheroid core and to detect and characterize cells outside this mask. We have developed two strategies for mask computation, one for compact spheroids and another for models that lose their boundaries soon after insertion into the extracellular matrix. In both modes, masks can be created for spheroids of various shapes. Cells or their clusters outside the mask are recognized on the basis of filtered local maxima. This method enables the analysis of images with a nonconstant background, which is often found in real fluorescence images. The evaluation is largely automated but allows visual inspection based on the overlay of the objects detected by the algorithm with the original fluorescence signal of the spheroid core and the invading cells. A user-friendly manual adjustment of the parameters for mask fitting and cell detection is implemented.

## Graphical abstract

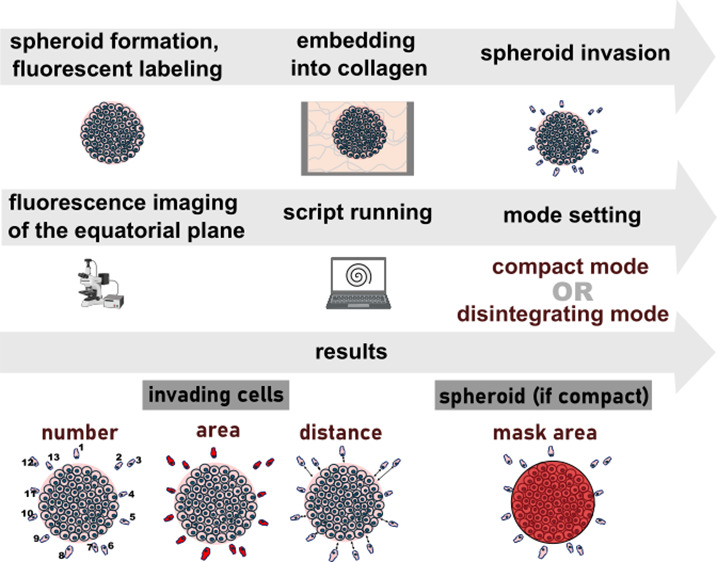

## Introduction

Metastasis is the most important factor influencing the 5-year survival rate for various types of cancer.^1,2^ Local invasion of cells into adjacent tissue is an important prerequisite for the metastatic cascade.^3,4^ Although extracranial metastases of glioblastoma are rare, the tumor is characterized by aggressive infiltration into the surrounding brain tissue.^5^ Current treatment options are often limited by the lack of robust preclinical cancer models. Compared to monolayers, three-dimensional (3D) models provide more accurate conditions that reflect the complex tissue environment of a tumor growing *in vivo*, including hypoxia, acidosis, and concentration gradients for nutrients. These 3D models include spheroids, which are derived from established cell lines, and organoids, which are derived from tumor tissue of each individual patient.^6,7^

One of the most important techniques for characterizing 3D models is to embed them in an extracellular matrix and then observe their growth and invasion into the surrounding matrix. This 3D invasion microenvironment is usually created using Matrigel or collagen.^8^ The growth and invasion of the spheroids are then determined using various microscopic techniques, including both bright-field^9^ and fluorescence imaging.^10^

Currently, it remains a challenge to analyze the resulting image data and to compare the invasive potential of cells in different samples. Automated approaches to identify areas with invasive cells using tools implemented in ImageJ can be helpful but are often unsuitable for evaluating irregularly shaped spheroids. In some cases, in-house developed macros and automated processes to quantify invasive cell areas have been proposed, but these solutions have not seen widespread adoption for analysis of larger sample sets.^11–14^

Therefore, this project introduces a semiautomatic algorithm to assess the growth and expansion area of fluorescently labeled spheroids, suitable for diverse spheroid shapes and invasion patterns. The detection of objects in the extracellular matrix is performed using robust approaches that are insensitive to fluctuations in background fluorescence and allow a reliable comparison of the expansion area among multiple samples.

## Experimental Section

### Spheroid Formation and Cultivation

The human osteosarcoma cell lines HOS and 143B (kindly provided by Bruno Fuchs), the human colorectal carcinoma cell lines HT-29 and HCT-116 from LGC Standards (Teddington, UK), and the human glioblastoma U-251 MG (ATTC) were cultured in Dulbecco’s Modified Eagle Medium (DMEM) (Sigma-Aldrich, St. Louis, Missouri) supplemented with 10% fetal bovine serum (FBS) (Invitrogen, Carlsbad, California), 2 mM l-glutamine, penicillin (100 U/mL), and streptomycin (100 U/mL) (Lonza, Basel, Switzerland) in a humidified incubator with 5% CO_2_ at 37 °C. U-251 MG cultures were additionally supplemented with MEM Non-Essential Amino Acid Solution (Sigma-Aldrich).

For spheroid formation, cells were detached using trypsin (Invitrogen) and seeded onto a 12-well plate coated with 1% agar in 1× phosphate-buffered saline (1 × PBS; Sigma-Aldrich) using DMEM supplemented with 2 mM l-glutamine, penicillin (100 U/mL), and streptomycin (100 U/mL) without FBS. Cells were seeded at densities adjusted to their growth characteristics: 143B, HT-29, and HCT-116 at 50,000 cells/mL; HOS at 100,000 cells/mL; and U-251 MG at 20,000 cells/mL. The 12-well plates containing the cells were placed on a horizontal rotary shaker (Orbital Shaker, NB-101SRC, N-BIOTEK, Korea) at 58 rpm and incubated in a humidified incubator with 5% CO_2_ at 37 °C for 24 h. The following day, the medium was supplemented with 10% FBS and the spheroids were incubated for a further 3 days. Three-day-old U-251 MG spheroids were treated with 0.1 and 1 μM triptolide (HY-32735; MedChem Express, Monmouth Junction, New Jersey, USA) or dimethyl sulfoxide (DMSO; Thermo Fisher Scientific, Waltham, Massachusetts, USA) as a solvent for triptolide. This treatment was performed 1 day prior to embedding the spheroids in collagen.

### Spheroid Staining

Two different spheroid staining protocols were used for thorough script testing to improve and optimize the protocol. Cell lines 143B and HOS were stained prior to spheroid formation. After detachment with trypsin, the cells were incubated for 30 min in FBS-free DMEM supplemented with 1 μM CellTracker Red CMTPX (Thermo Fisher Scientific). CellTracker-stained cells were used for the abovementioned spheroid formation. In the second protocol, 4-day-old spheroids formed from HT-29 and HCT-116 cells and from triptolide-treated U-251 MG cells were stained by incubation in FBS-free DMEM supplemented with 1 μM CellTracker Red CMTPX for 60 min.

### Collagen Embedding

Four-day-old stained spheroids were embedded in 100 μL of collagen (adjusted to pH ∼ 7 with 1 M NaOH) mixed with 10 μL of 10× concentrated DMEM in a 96-well plate previously coated with 1% agar in 1× PBS. The embedded spheroids were cultured in a humidified incubator with 5% CO_2_ at 37 °C for 1 h to facilitate collagen stiffening. Subsequently, 50 μL of DMEM supplemented with 10% FBS, 2 mM l-glutamine, penicillin (100 U/mL), and streptomycin (100 U/mL) were carefully added over the gel. In experiments with U-251 MG, DMSO or triptolide was added directly into both the collagen mixture and the medium overlaying the gel. The embedded spheroids were incubated in a humidified incubator with 5% CO_2_ at 37 °C for various periods of time before being microscopically inspected.

### Microscopy

Fluorescence and bright-field images were captured using a Leica 6000B widefield microscope; image acquisition was performed with LAS X (Version 3.5.7.23225) (Leica Microsystems, Germany). The spheroids were stained with CellTracker Red CMTPX, which has excitation and emission maxima at 577 and 602 nm, respectively. The excitation and emission filters, as well as the dichroic mirror, were configured as follows: EX: 515–561 nm; DC: 580 nm; EM: 590 nm. The optical magnification was set to a total of 50× using a 5× Leica Fluotar objective (Leica Microsystems) with field planarity. 2D images were taken by focusing on the single equatorial plane of the spheroid to visualize both the spheroid and the invading cells within the collagen gel. Fluorescence images were taken immediately after embedding and solidification of the matrix (START) and at subsequent time points (24, 48, 72, and 96 h). The .lif image sequences were imported into ImageJ 1.54f (National Institutes of Health, USA) and processed using a newly developed algorithm.

### Statistics

Statistical analysis was performed using OriginPro 2023 software (OriginLab Corporation, Northampton, Massachusetts, USA). Friedman′s ANOVA was used to analyze multiple paired groups, followed by Dunn’s post hoc analysis for pairwise comparisons. Kruskal–Wallis ANOVA with Dunn’s post hoc analysis was used for multiple unpaired comparisons. Comparisons between two groups were performed using the Wilcoxon signed-rank test for paired data and the Mann–Whitney *U* test for unpaired data.

Data are presented as mean, median line, range with 1.5 IQR (interquartile range), 25–75% interval, and outliers. At least three independent experiments were performed.

## Results and Discussion

In this project, an algorithm for the quantification of cell expansion from 3D tumor models into the extracellular matrix was developed. After loading the data set, which consists of the starting image and the images of the subsequent time intervals, the macro was executed. Upon initialization, the user is presented with a dialogue window (Figure 1A). In the upper part of the dialogue window, the user can choose the name of the starting image (“Starting image” field) and then define the entire set of images for analysis (“Select images to analyze” section). The evaluation of the invasive potential followed a general scheme involving the identification of the compact spheroid core, computation of its mask, and the subsequent detection, quantification, and analysis of objects outside the mask, excluding potential artifacts.

**Figure 1:**
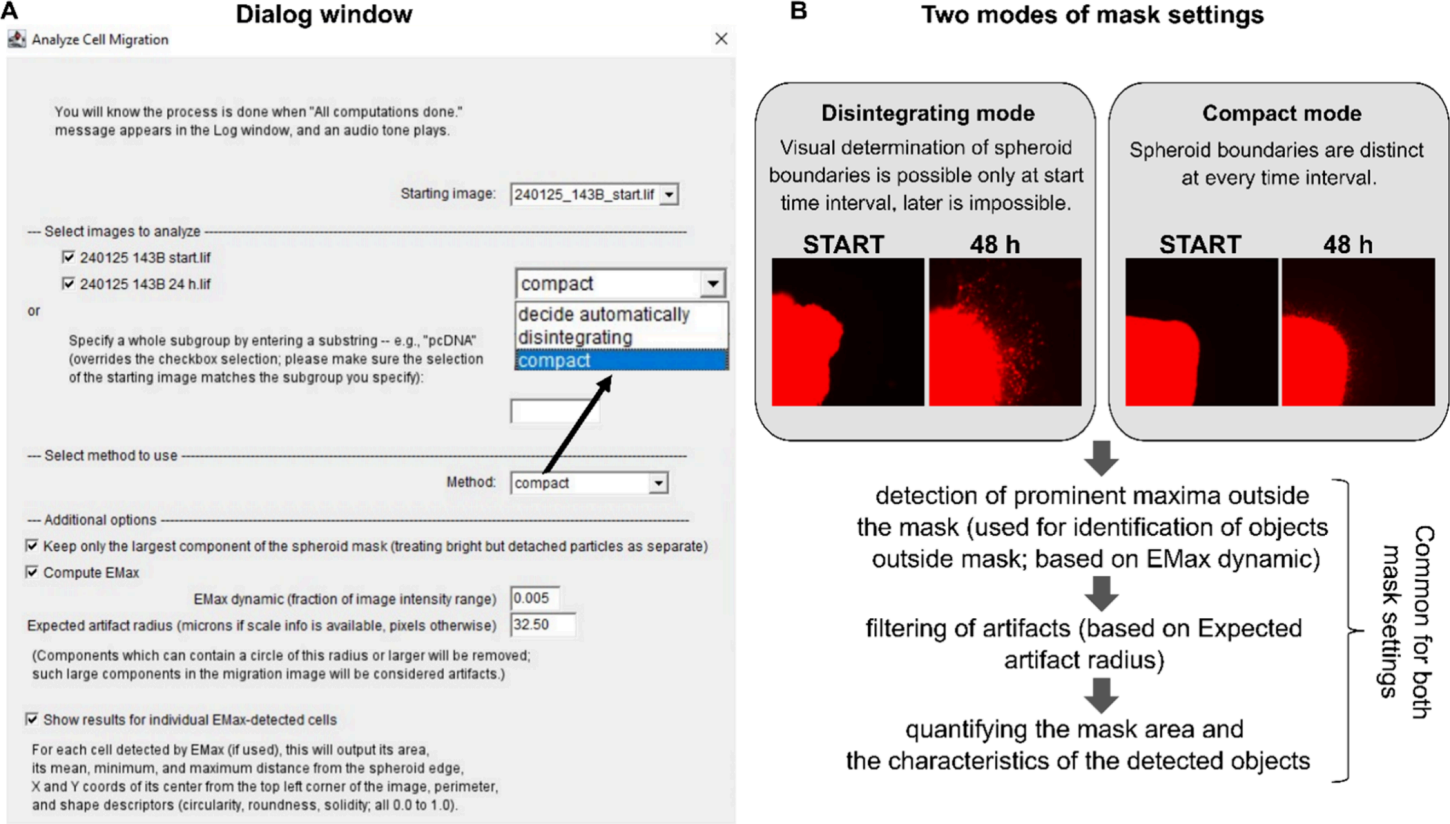
(A) Dialog window allowing the user to specify the complete set of images for analysis, including the starting image. The method of image analysis can be selected (“Disintegrating” mode, “Compact” mode, “Decide Automatically”). The option to detect only the largest compact mask component is selected by default. Fields for the values of EMax dynamic and Expected artifact radius are shown. (B) Overall diagram of the algorithm. The modes used for mask generation are summarized together with the morphological properties of the spheroids that are critical for determining whether the “Disintegrating” or “Compact” mode is selected.

The mask is calculated in two modes depending on the compactness of the model: one for disintegrating spheroids and another for compact spheroids. If the appropriate mode is unclear, the algorithm can automatically determine the most suitable option (Figure 1A; “Method” field). Potential disintegration of the spheroid core over time can be taken into account by activating the option “Keep only the largest component of the spheroid mask (treating bright but detached particles as separate)”. The identification of cells outside the mask is allowed by the parameters “EMax dynamic” and “Expected artifact radius” (Figure 1A,B). The detailed table with results and its summary are displayed after the computation, as described below.

### “Disintegrating” Mode

The mode for rapidly invading spheroids is suitable for models that retain their compact structure at the START time interval immediately after insertion into the extracellular matrix, but whose boundaries are then rapidly lost due to their invasive properties (Figure 1B).

In this workflow, Minimum Cross Entropy thresholding^15^ of the fluorescently labeled spheroid from the background is applied at the START time interval. This segmentation generates a mask whose area is calculated (Figure 2A). The mask **o** **f** **t** **h** **e** **s** **a** **m** **e** **a** **r** **e** **a** is then applied to the images captured at later time intervals, covering the highest-intensity pixels of the image (Figure 2B). Objects that lie outside the area are considered as invading cells. A critical assumption underlying this mode is that the individual cells invading out of the spheroids are less fluorescent than the original spheroid core due to their separation.

**Figure 2:**
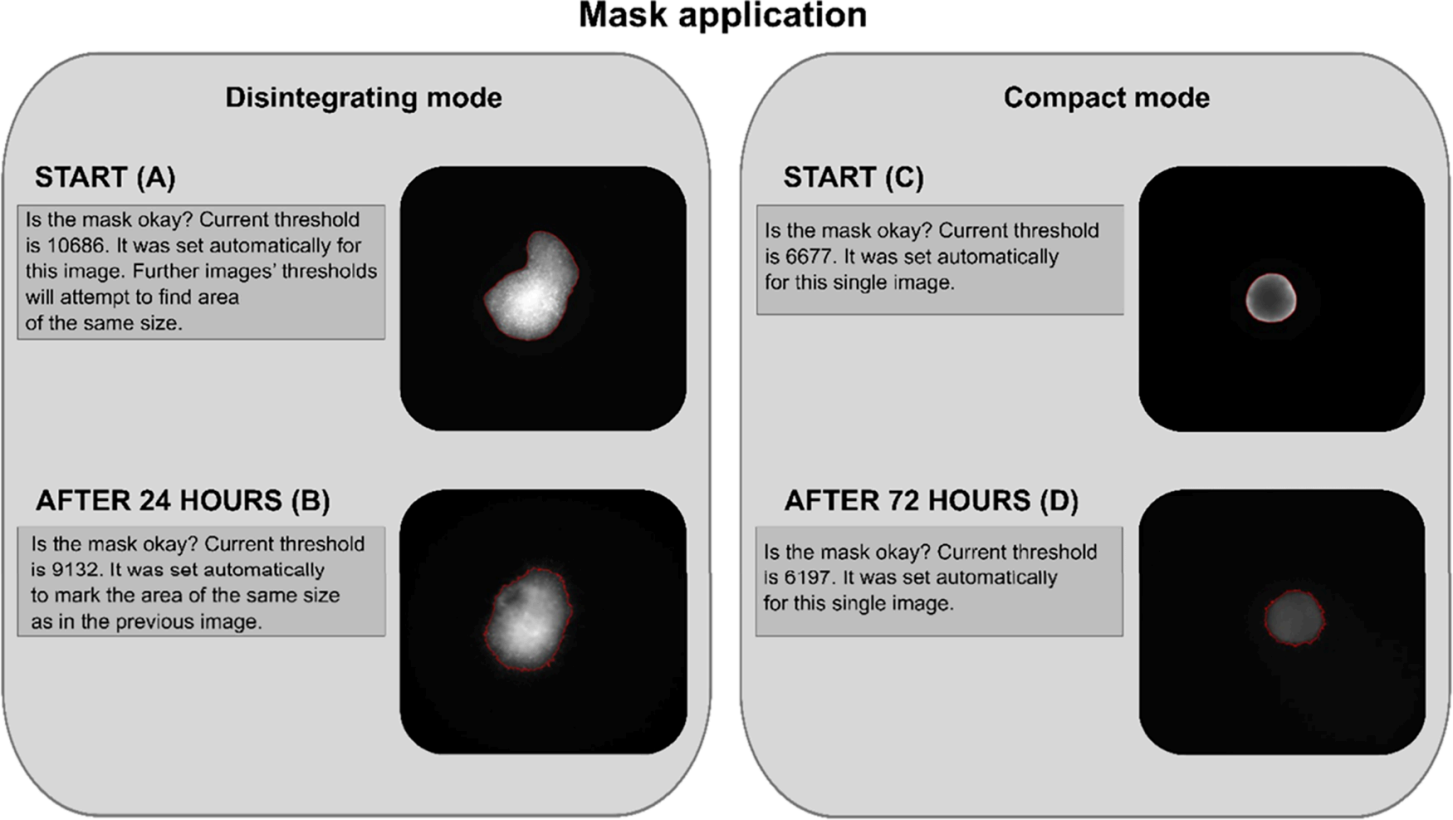
Mask calculation for START and 24 h time intervals. For the “Disintegrating” mode, the mask is generated by thresholding fluorescent spheroids from the background at the START time point. The mask area is calculated (A). At the 24 h time interval, a mask of the same size is created (B). For the “Compact” mode, thresholding for mask generation is used both at the START time (C) and at the 72 h interval (D). The mask boundaries (in red) are visualized in an overlay with the fluorescent spheroid (A–D).

If the spheroid core area decreases at later time points compared to the starting time point—indicating rapid disintegration—then only the largest **compact** mask component should be retained for further analysis. This is ensured by selecting the option “Keep only the largest component of the spheroid mask”. If this option is not selected, then the mask may mistakenly include detached objects that are no longer part of the spheroid core (Supporting Figure S1).

### “Compact” Mode

The “Compact” mode is based on maintaining the distinct contrast between the spheroid boundary and the background throughout the entire experiment (Figure 1B). The masks are computed by thresholding between the fluorescent spheroid and the background not only at START but also at the following time intervals (Figure 2C,D). No information is transferred from the START image to the subsequent time points. This method was implemented after recognizing that the “Disintegrating” mode is unsuitable for spheroids that grow compactly and increase their area over time without disintegration (Supporting Figure S2). If the “Disintegrating” mode was incorrectly selected for the analysis of compactly growing spheroids, then the mask fitting can be optimized by adjusting the thresholding parameters (Supporting Figure S2).

In both modes, users can verify the mask accuracy. To ensure accurate detection, the fluorescence image overlay with the suggested mask boundaries is provided by the algorithm (Figure 2A–D). If the mask does not match the core of the spheroid, the user can manually adjust the thresholding parameter in the pop-up window, regenerate the mask, and check the overlay again to confirm the correct match before proceeding with further analysis. This verification loop can be repeated until the user is satisfied with the mask adjustment.

Identification of the objects outside the mask is done using the Extended Maxima transformation (EMax), detecting local maxima that meet a user-defined contrast criterion between them and their surrounding area. The contrast criterion is specified as a fraction of the image intensity range. The value of this fraction (a default value is provided) must be entered in the “EMax dynamic” field in the Additional options (Figure 1A) after the “Compute EMax” option has been activated. To distinguish cells from potential contaminants, bubbles, and background fluctuations, the fluorescent objects outside the mask are displayed in an overlay with the identified objects both in the START (Figure 3A and Supporting Figure S3) and the subsequent time interval (Figure 3B and Supporting Figure S3; identified objects in green). For better alignment, it is possible to switch interactively between this overlay and the fluorescence image alone (Figure 3; arrows). It is recommended to use the lowest EMax dynamic value that is high enough to detect the smallest possible number of objects outside the mask at the START time, when no migration should have taken place. No invading cells should be outside the mask at the start, and any detected objects may be contaminants or bubbles that have been transferred with the spheroid into the extracellular matrix. In one example, at Emax dynamic 0.0001, thousands of objects may be detected outside the START mask, whereas at dynamic values of 0.001 and 0.01, seven objects and one object may be detected, respectively (Supporting Figure S3). Thus, both EMax dynamics of 0.001 or 0.01 appear to be suitable. To select the optimal dynamic value, it should be low enough to detect as many objects as possible outside the mask at subsequent time intervals. The interactive overlay visualization helps with this decision making. An EMax dynamic value of 0.001 is more suitable as it detects almost all objects outside the mask (644 objects with an EMax dynamic value of 0.001 compared to 87 objects with a dynamic value of 0.01; Supporting Figure S3). Once selected, this dynamic value should be used for all evaluations of the same data set.

**Figure 3:**
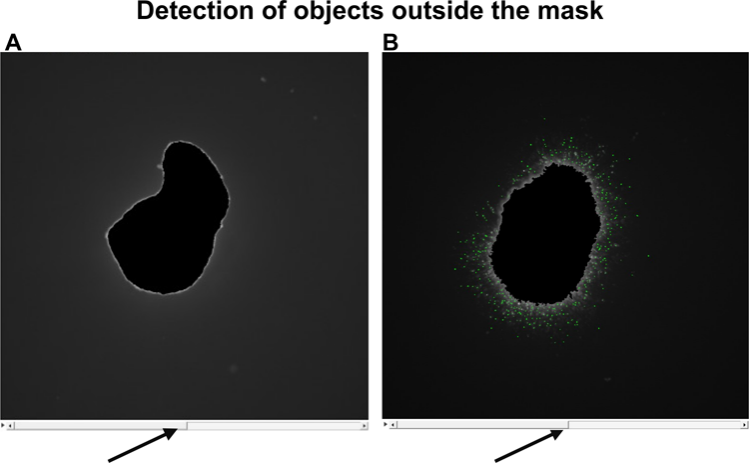
Detection of objects outside the masks at START (A) and after 24 h (B). Overlays of detected objects (in green) with the corresponding fluorescence images are shown. Interactive switching (arrows) between overlays and fluorescence images enables direct comparison.

The Artifact exclusion is based on the object size, as the available images can sometimes contain artifacts such as bubbles or background fluctuations whose size exceeds that of the cells. This is achieved by entering the expected artifact radius in microns into the corresponding field in the Additional options (Figure 1A). All detected objects outside the mask that can contain a disk of the specified radius are excluded from the analysis. The exclusion of artifacts can be affected not only by the setting of the Expected artifact radius but also by the choice of the appropriate EMax dynamic value or by defining the region of interest (Supporting Figure S4).

The output includes a summary with the mask area and perimeter, the number of objects outside the mask (in total and per unit of length of the perimeter), the total area of the objects outside the mask, and the mean and median distances of the objects to the nearest pixel on the spheroid boundary (Figure 4). To calculate the perimeter, the spheroid mask is smoothed to contain only details larger than ∼10–20 μm.

**Figure 4:**
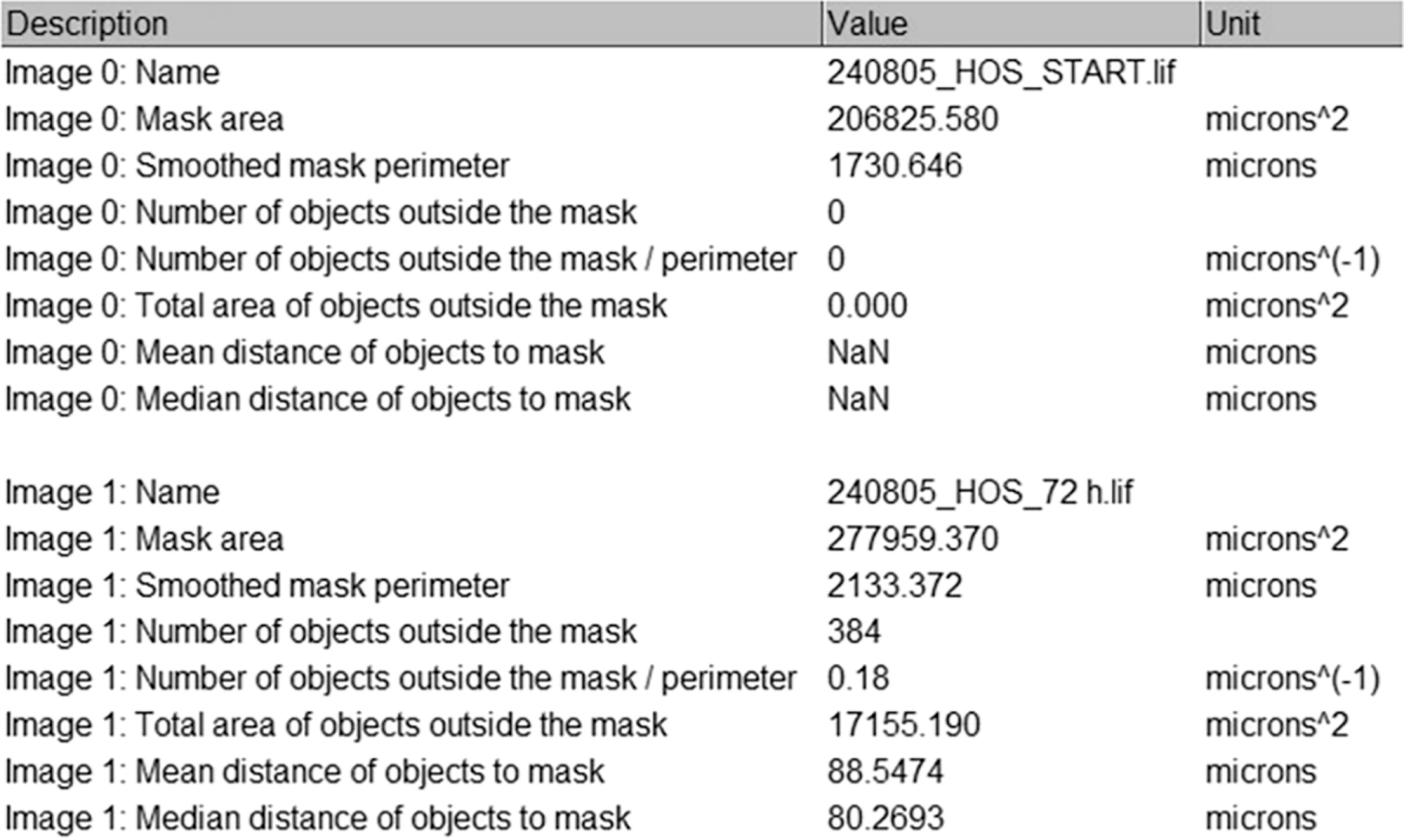
Summary table showing the mask area, the mask perimeter, the number of objects outside the mask (in total and per unit of length of the mask perimeter), their total area, and the mean and median distances to the nearest edge of the mask.

Further details on the characteristics of the entire image, the mask, and the detected objects are shown in the Results table. This includes for each detected object the position, the distance to the nearest point of the mask, the area, the perimeter, and the shape descriptors (circularity, roundness, and solidity). Further filtering can be performed based on these characteristics.

For the purpose of this article, spheroid growth refers to an increase in the area of the compact spheroid core defined by the mask calculated by the algorithm. When spheroids lose their compact structure during the experiment, the calculated mask size is set at START and remains constant in the following intervals. Therefore, growth is not applicable to these spheroids.

In contrast, the term spheroid expansion applies to both compact spheroids and spheroids losing compactness and refers to the increase in the number and area of objects located outside the spheroid mask, regardless of the underlying biological processes—whether driven by invasion, proliferation, or a combination of both.

The functionality of the algorithm was tested on HOS and 143B cell lines. The 143B cell line was derived from HOS cells by K-Ras transformation; therefore, its invasive potential is increased compared to HOS.^16^ Spheroids cultured from 143B and HOS cells were analyzed using the “Disintegrating” or “Compact” modes, respectively, to reflect differences in their invasive behavior. The algorithm detected expansion of both HOS- and 143B-derived spheroids, based on the quantification of the number and area of objects located outside the mask (Figure 5A–C). As expected, the extent of expansion was greater for 143B spheroids than for HOS spheroids (Figure 5A–C). At the 24 h time point, the mean and median distances of expanding objects from 143B spheroids were greater than those from HOS spheroids (Figure 5D). Script-generated visualizations illustrating the mask application and the objects outside the mask are shown in Figure 5E. These findings demonstrate that the algorithm is able to distinguish differences in spheroid expansion between 143B and HOS models.

**Figure 5:**
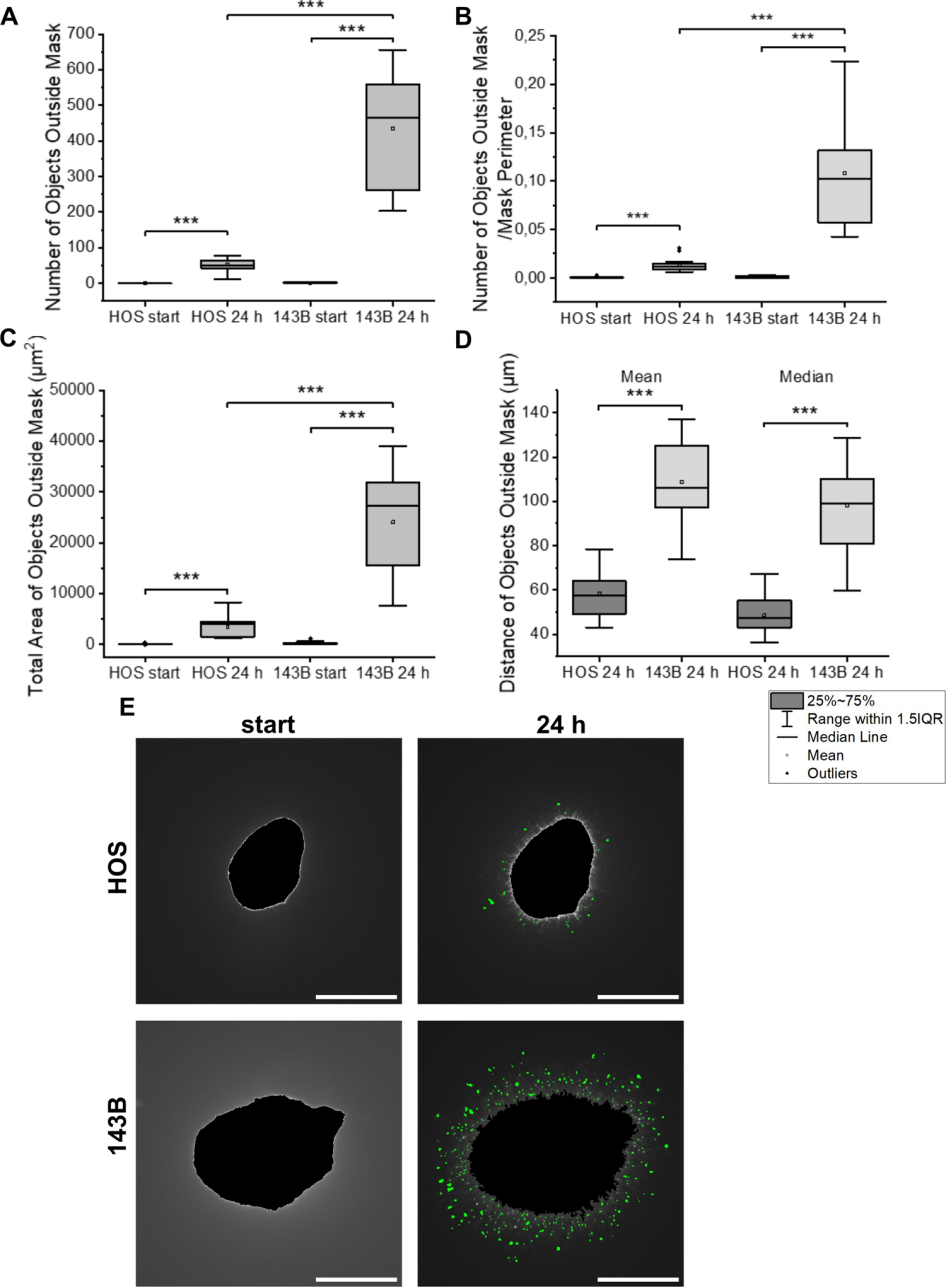
HOS- and 143B-derived spheroids were embedded in a collagen matrix. Expansion into the extracellular matrix was quantified using the number (in total and per unit of the length of the mask perimeter) and area of objects outside the mask (A–C). Their mean and median distances from the nearest point on the spheroid boundary (D) were also determined. Significant comparisons are indicated by asterisk brackets; *p < 0.05, **p < 0.01, ***p < 0.001. Wilcoxon signed-rank test was used for HOS vs HOS 24 h and 143B vs 143B 24 h data; Mann–Whitney *U* test for the HOS vs 143B comparison. Representative images of objects (green) detected outside the mask (black) in spheroids derived from HOS and 143B cells at START and after 24 h, scale bar 500 μm (E).

Next, we analyzed the HOS spheroids at START, 24, 48, and 72 h intervals using the “Compact” mode. Over time, we observed an increase of spheroid core area, indicating spheroid growth (Figure 6A), as well as an increase in the number and total area of objects outside the mask, and their mean and median distances from the spheroid core (Figure 6B–F). Representative images of spheroid growth and expansion over time are shown in Figure 6G.

**Figure 6:**
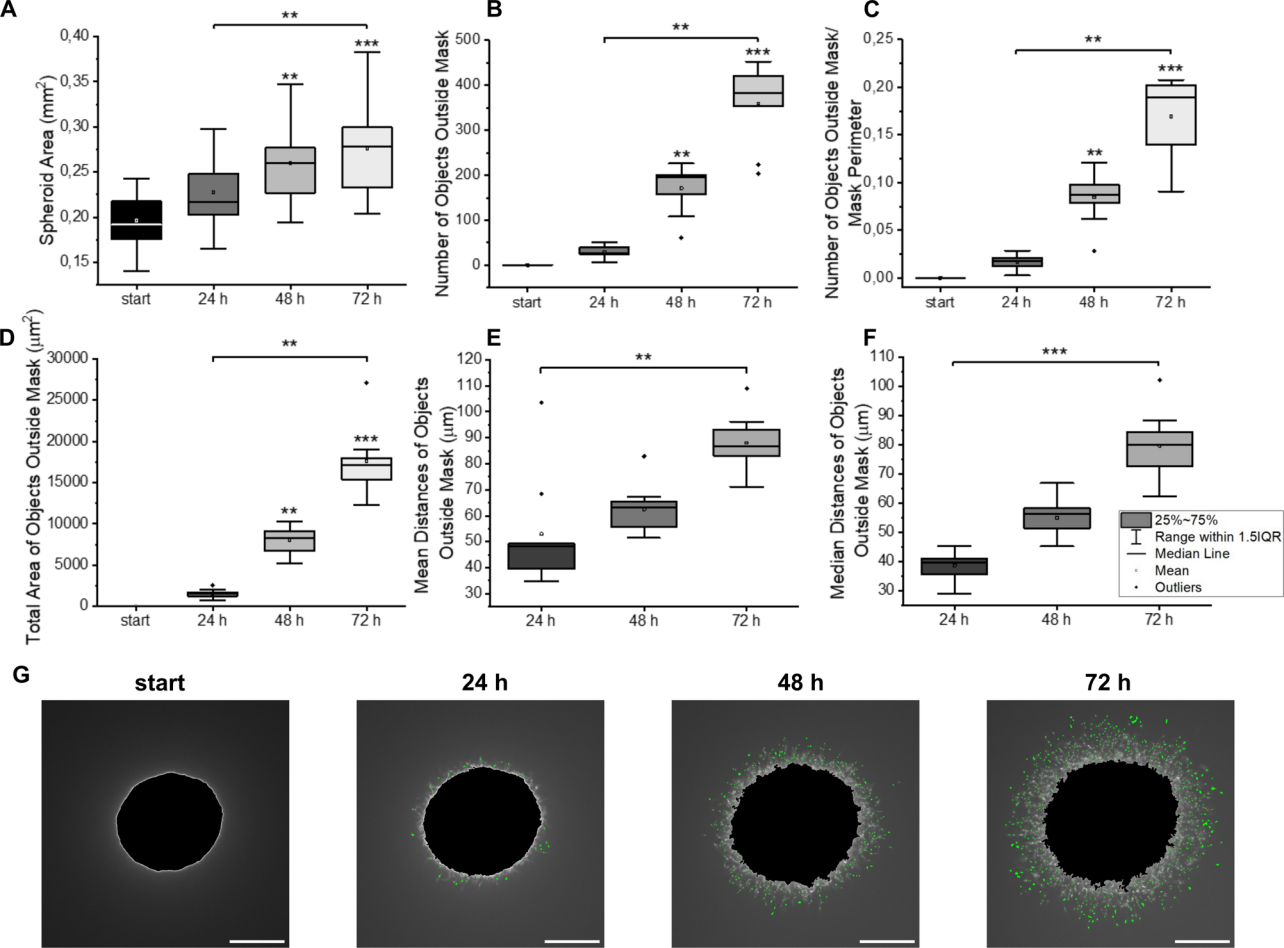
HOS-derived spheroids were embedded in a collagen matrix. Their growth was assessed for 3 days based on the spheroid area (A). Expansion into the extracellular matrix was quantified using the number of objects outside the mask (in total and per unit of the length of the mask perimeter) and their total area. Mean and median distances of the objects outside the mask from the nearest point of the spheroid boundary were also assessed (B–F). Significant differences between START and following time intervals as determined by Friedman′s ANOVA are marked with asterisks; other significant comparisons are indicated by asterisk brackets; *p < 0.05, **p < 0.01, ***p < 0.001. Representative images of objects (green) detected outside mask (black) in spheroids at START and after 24, 48, and 72 h, scale bar 250 μm (G).

To demonstrate the broad applicability of the script, we performed experiments with triptolide, an inhibitor of glioblastoma (GBM) migration and invasion.^17^ At 72 h after embedding, triptolide at a dose of 1 μM decreased the number of objects outside the mask in U-251 MG-derived spheroids compared to both controls and the 0.1 μM dose, as assessed using the “Compact” mode (Figure 7A). Moreover, we recorded a notable decrease in the normalized number of objects outside the mask relative to the mask perimeter (Figure 7B), their total area (Figure 7C), and the mean distance from the spheroid core (Figure 7D), with significant reductions at both 0.1 and 1 μM concentrations compared to controls. Script-generated images documenting these findings are shown in Figure 7E. These results demonstrate the potential of the algorithm to characterize the effects of drug treatment on glioblastoma spheroid expansion.

**Figure 7:**
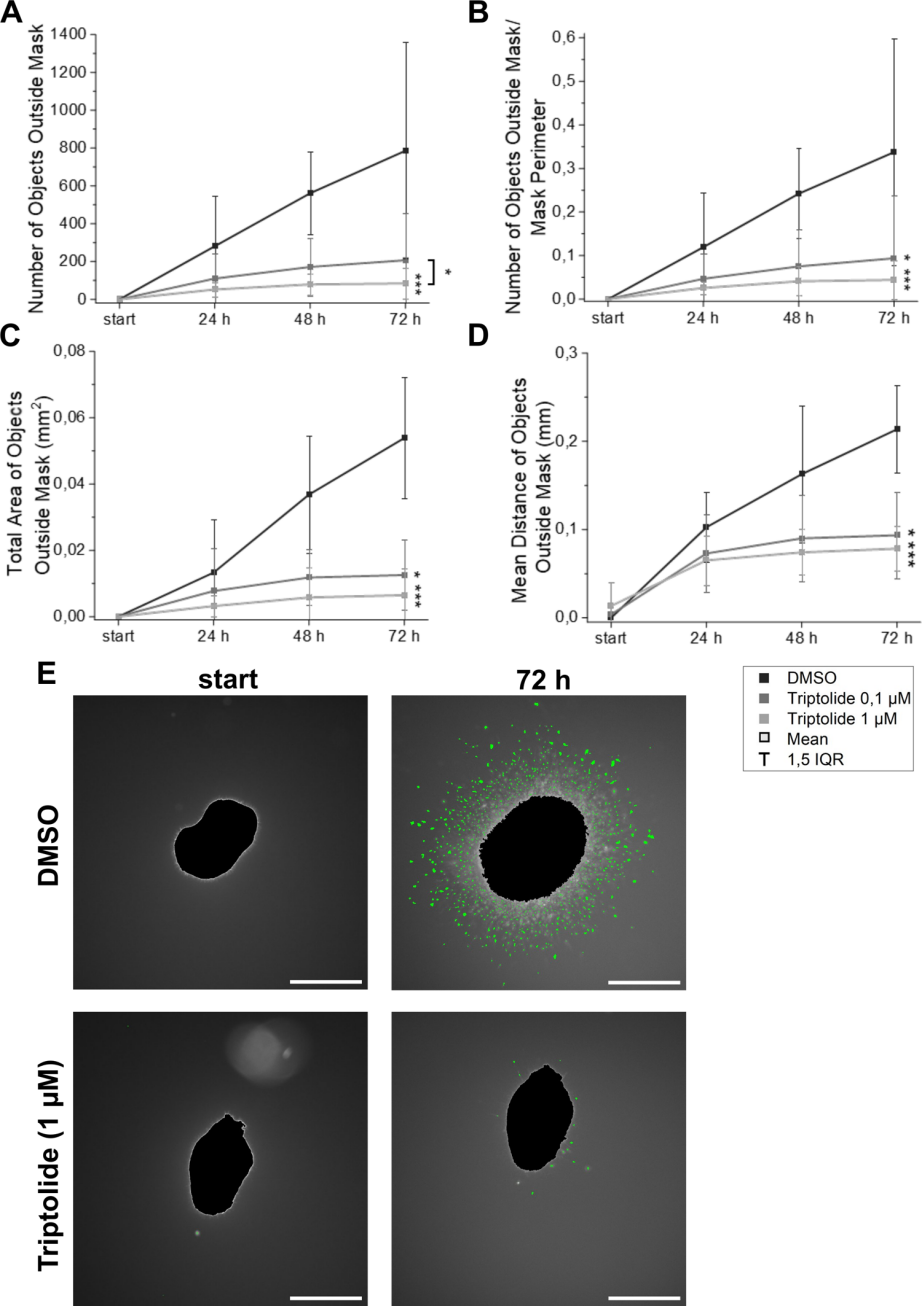
Spheroids derived from U-251 MG cells pretreated with triptolide and DMSO-treated controls were embedded in collagen gel. The number of objects outside the mask (total and normalized per unit length of the mask perimeter), their total area, and the mean distance from the nearest point on the spheroid boundary were quantified (A–D). Significant differences between controls and triptolide-induced samples determined by Kruskal–Wallis ANOVA are indicated by asterisks. Additional significant comparisons are shown with asterisk brackets; *p < 0.05, **p < 0.01, ***p < 0.001. Representative images of objects (green) detected outside the mask (black) in the controls and the triptolide-treated spheroids at START and after 72 h, scale bar 500 μm (E).

To further test the functionality of the script, we examined the growth of spheroids derived from the HT-29 and HCT-116 cell lines, the former being less invasive than the latter.^18^ In both cases, the spheroid area increased over time (Supporting Figure S5A,B). The number of objects outside the mask increased over time only for HCT-116 spheroids (Supporting Figure S5C–F).

### The Advantages of the Method

1. Our method uses fluorescence images, which are clearer and less affected by artifacts than bright-field images used in some other approaches.^11,19,20^

2. The mask can be computed for spheroids of various shapes. Other biological models, such as tumoroids or organoids, can also be evaluated, as long as their morphology visually resembles the spheroids described in this article.
3. The algorithm allows the evaluation of both spheroids with clear boundaries and spheroids that have lost sharp boundaries during the course of the experiment, selecting the appropriate mode automatically.
4. The evaluation is largely automated, reducing intra- and interpersonal variance, but it allows the user to easily adjust key parameters (especially thresholds) based on a visual assessment of the image data in contrast to manual methods reported by other groups.^11,21^

5. The algorithm enables reliable detection of objects outside the mask, even in images that do not have a constant background value.
6. Since individual objects are detected (as opposed to just their total area, as described in previous studies^10,20^ ), we are able to evaluate their detailed characteristics, such as the distance migrated from the spheroid boundary, as demonstrated in Figures 4–7.
7. The computation of shape descriptors enables further filtering of detected objects based on their shape.

In the case of rapidly invading spheroids with unstable morphology, the exact boundary is no longer clearly recognizable. This precludes both a simple segmentation of the spheroid and the use of a fixed-shape mask throughout the experiment. By computing a mask that covers the area of the same size as at START but allows its shape to evolve, these morphological changes are taken into account. The distances of the migrating cells are then measured from the boundary of this arbitrarily shaped mask, giving more accurate results than, e.g., measuring them from its centroid as done by other methods,^13^ particularly in the case the mask is irregular, elongated, or even with lobes or protrusions. When the spheroid actually exhibits a highly dispersive phenotype, only the largest compact component of mask is taken for further analyses to ensure that all detached objects are considered as invading.

To make the results of spheroids of different sizes comparable, the number of cells outside the mask is normalized by the perimeter of the spheroid mask. This perimeter is computed with a fixed degree of smoothing to ensure it is not influenced by small protrusions.

### The Disadvantages of the Method

Identification of objects outside the mask based on local maxima does not correspond to an exact object segmentation. In the case of a cell with several distinct intensity maxima, this single cell may be recognized as several objects, or a cluster of cells may be identified as only a single object, depending on the EMax dynamic parameter. The algorithm focuses on detection rather than full segmentation, and therefore the detected maxima may not cover the entire fluorescent area of the objects.

Another important aspect is that no proliferation inhibitor was added to the collagen matrix. Therefore, differences in the characteristics of objects outside the mask may result from both the invasion of cells from the spheroid core and their proliferation. For this reason, the term “spheroid expansion” rather than “invasion” is used in this project.

## Conclusions

We have presented a new method for analysis of 3D tissue models, such as spheroids embedded in extracellular matrix, which can be used to characterize their invasive potential and growth. We discussed the advantages compared to existing approaches, namely, the degree of automation and the diversity of invasion-related measurements. Another distinct feature of our algorithm is its ability to analyze both compact spheroids and disintegrating spheroids without a clear boundary.

## Supplementary Material



## Data Availability

The ImageJ script and a sample data set for testing are publicly available at https://gitlab.fi.muni.cz/cbia/cellinvasion.

## Notes

(Figure S1) Effect of “Keep only the largest component of the spheroid mask” option; (Figure S2) impact of incorrect mode selection (“Disintegrating” vs “Compact”); (Figure S3) optimization of Emax dynamic value for object detection; (Figure S4) artifact exclusion; and (Figure S5) growth and expansion of HT-29 and HCT-116 spheroids (PDF)

## References

[1] Odri G. A., Tchicaya-Bouanga J., Yoon D. J. Y., Modrowski D. (2022). Metastatic
Progression of Osteosarcomas: A Review of Current Knowledge of Environmental
versus Oncogenic Drivers. Cancers..

[2] Shin A. E., Giancotti F. G., Rustgi A. K. (2023). Metastatic colorectal cancer: mechanisms
and emerging therapeutics. Trends Pharmacol.
Sci..

[3] Pretzsch E., Bösch F., Neumann J. (2019). Mechanisms
of Metastasis
in Colorectal Cancer and Metastatic Organotropism: Hematogenous versus
Peritoneal Spread. J. Oncol..

[4] Sheng G., Gao Y., Yang Y., Wu H. (2021). Osteosarcoma and Metastasis. Front. Oncol..

[5] Vollmann-Zwerenz A., Leidgens V., Feliciello G., Klein C. A., Hau P. (2020). Tumor Cell
Invasion in Glioblastoma. Int. J. Mol. Sci..

[6] Guan X., Huang S. (2022). Advances in
the application of 3D tumor models in precision oncology
and drug screening. Front Bioeng Biotechnol..

[7] Meijer T. G., Naipal K. A., Jager A., Van Gent D. C. (2017). Ex Vivo Tumor Culture
Systems for Functional Drug Testing and Therapy Response Prediction. Future Sci. OA.

[8] Bouchalova P., Bouchal P. (2022). Current methods for studying metastatic potential of
tumor cells. Cancer Cell Int..

[9] Vinci M., Box C., Eccles S. A. (2015). Three-Dimensional
(3D) Tumor Spheroid Invasion Assay. J. Vis Exp.

[10] Heiss J., Tavana H. (2024). Automated Analysis
of Extracellular Matrix Invasion
of Cancer Cells from Tumor Spheroids. ACS Meas
Sci. Au..

[11] Berens E. B., Holy J. M., Riegel A. T., Wellstein A. (2015). A Cancer Cell
Spheroid Assay to Assess Invasion in a 3D Setting. J. Vis Exp.

[12] Evensen N. A., Li J., Yang J. (2013). Development of a High-Throughput Three-Dimensional
Invasion Assay for Anti-Cancer Drug Discovery. PLoS One.

[13] Mei J., Böhland C., Geiger A. (2021). Development of a model
for fibroblast-led collective migration from breast cancer cell spheroids
to study radiation effects on invasiveness. Radiat Oncol..

[14] De
Wever O., Hendrix A., De Boeck A. (2010). Modeling
and quantification of cancer cell invasion through collagen type I
matrices. Int. J. Dev Biol..

[15] Li C. H., Lee C. K. (1993). Minimum cross entropy
thresholding. Pattern Recognit..

[16] Říhová K., Dúcka M., Zambo I. S. (2022). Transcription factor
c-Myb: novel prognostic factor in osteosarcoma. Clin Exp Metastasis..

[17] Lai M., Liu L., Zhu L. (2021). Triptolide reverses epithelial-mesenchymal
transition in glioma cells via inducing autophagy. Ann. Transl Med..

[18] Bhattacharya A., Saluja S., Managuli V. (2021). Comparing Migratory
and Mechanical Properties of Human Bone Marrow-Derived Mesenchymal
Stem Cells with Colon Cancer Cells In Vitro. J. Gastrointest Cancer..

[19] Lim G. J., Kang S. J., Lee J. Y. (2020). Novel invasion
indices quantify the
feed-forward facilitation of tumor invasion by macrophages. Sci. Rep..

[20] Roper S. J., Coyle B. (2022). Establishing an In Vitro 3D Spheroid
Model to Study Medulloblastoma
Drug Response and Tumor Dissemination. Curr.
Protoc..

[21] Shabalina E. Y., Skorova E. Y., Chudakova D. A. (2021). The matrix-dependent
3D spheroid model of the migration of non-small cell lung cancer:
a step towards a rapid automated screening. Front Mol. Biosci..

