# Supplementary material for: Cell Invasion Analysis of Tumor Spheroids Using 2D Image Data
Source: ACS Meas Sci Au. 2025 Nov 27;6(1):59–67. doi: 10.1021/acsmeasuresciau.5c00121 (PMC12921606; doi:10.1021/acsmeasuresciau.5c00121)
Supplement: Supplementary file 1 [file tg5c00121_si_001.pdf]

Cell Invasion Analysis of Tumor Spheroids using 2D Image Data

Matěj Přikryl[1], Andrea Rousová[1], Ivana Acimovic[2], Petr Vaňhara[2,4], Lukáš Jan [2], Petr Beneš[1], Jan Šmarda[1], Michal Kozubek[3], Karel Štěpka,[3*] and Jarmila Navrátilová[1,4 *]

[1]Department of Experimental Biology, Faculty of Science, Masaryk University, 625 00 Brno, Czech Republic

[2]Department of Histology and Embryology, Faculty of Medicine, Masaryk University, 625 00, Brno, Czech Republic

[3]Centre for Biomedical Image Analysis, Department of Visual Computing, Faculty of Informatics, Masaryk University, 602 00 Brno, Czech Republic

[4]International Clinical Research Center, St. Anne´s University Hospital Brno, 602 00 Brno, Czech Republic

*To whom correspondence should be addressed:

Karel Štěpka, Ph.D.

Email: 172454@mail.muni.cz

Jarmila Navrátilová, Ph.D.

Email: jnavratilova@sci.muni.cz

ORCID NUMBERS

Matěj Přikryl: 0009-0003-2505-6764

Andrea Rousová: 0009-0002-3553-2499

Ivana Acimovic: 0000-0001-6992-3274

Petr Vaňhara: 0000-0002-7470-177X

Lukáš Jan: 0009-0007-5624-2200

Petr Beneš: 0000-0002-8297-9675

Jan Šmarda: 0009-0007-0960-5347

Michal Kozubek: 0000-0001-7902-589X

Karel Štěpka: 0000-0002-5711-6704

Jarmila Navrátilová: 0000-0002-3329-3108

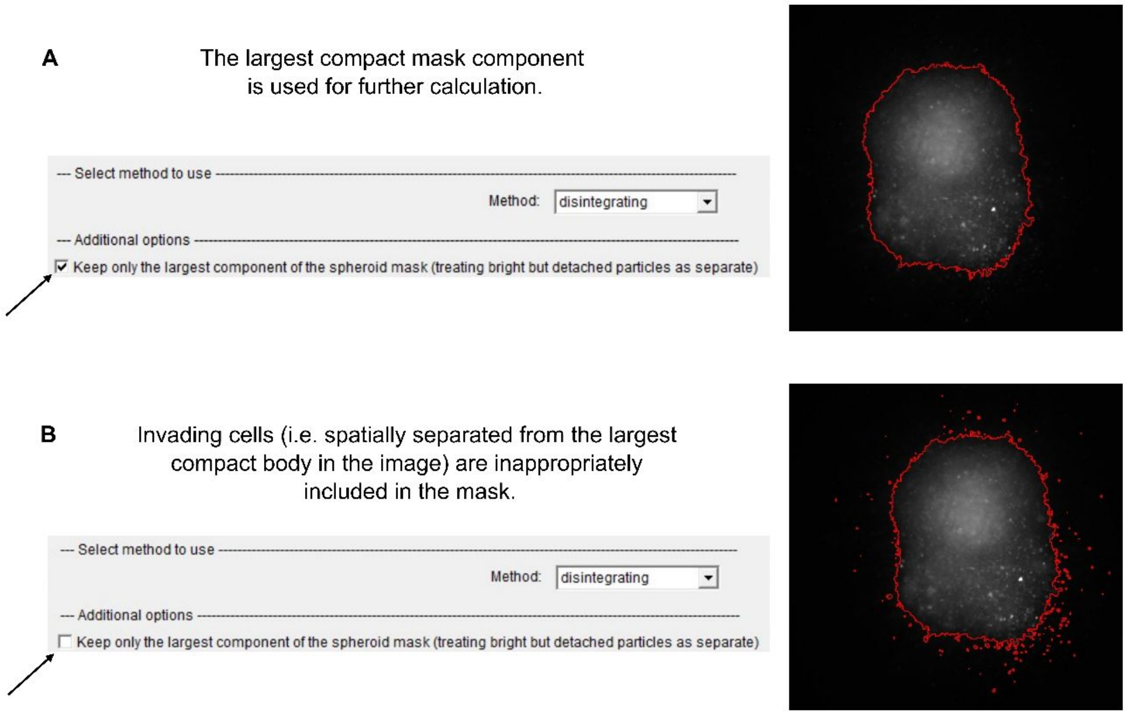

Figure S1

In "Disintegrating" mode, selecting the "Keep only the largest component of the spheroid mask" option ensures that all disconnected objects are excluded from the mask calculation (A). It is recommended to enable this option, as leaving it unchecked may mistakenly include spatially separate objects in the mask (B).

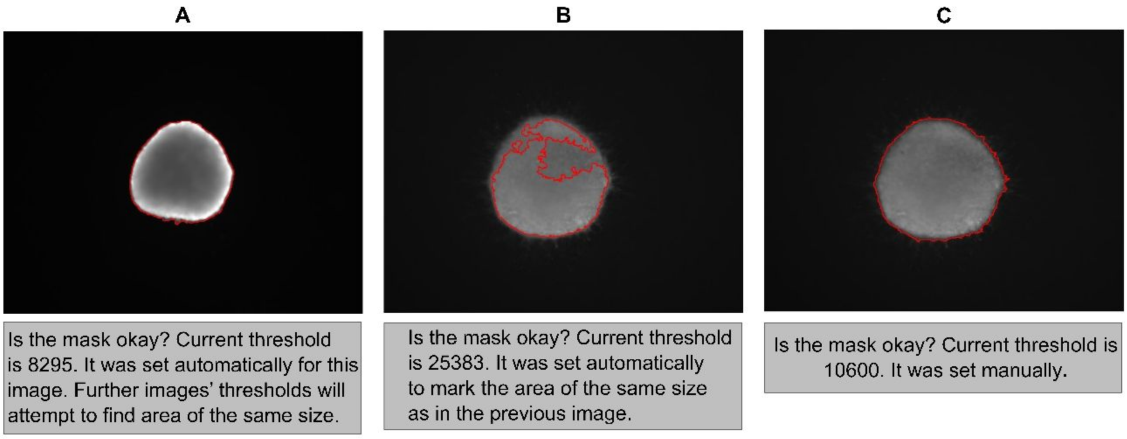

Figure S2

The "Disintegrating" method was mistakenly selected instead of "Compact" mode. As a result, the mask marking the same area as calculated at START (A) does not align with the spheroid boundaries at the later time point (B). This misalignment leads to a mask area smaller than the spheroid core, as seen from the visual inspection of the fluorescent spheroid core overlaid with the mask boundary. However, since the spheroid boundaries are well-defined, the threshold can be either manually adjusted to correct the mask fitting (C), or more appropriately, the "Compact" mode can be used (as suggested for this image set by the automatic mode selection).

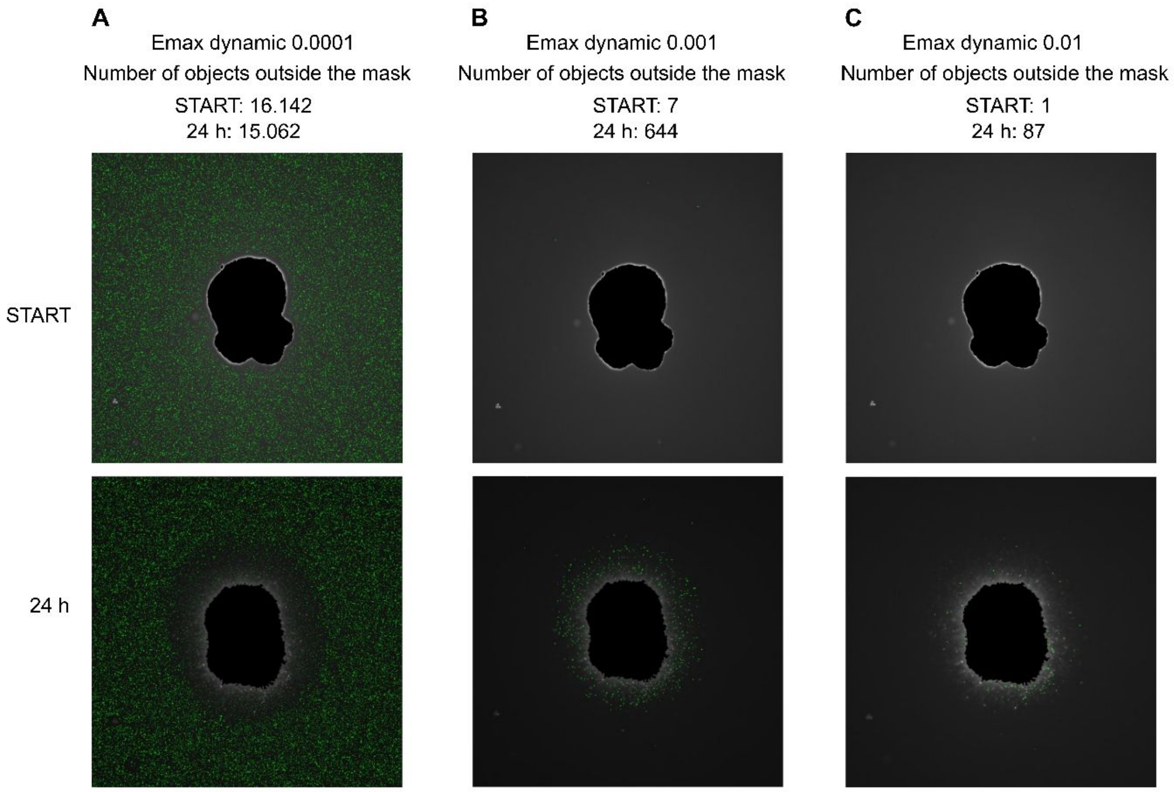

Figure S3

The relationship between the EMax dynamic value and the number of objects detected outside the mask at START and 24 hours. Overlays of detected objects (in green) and the corresponding fluorescence images are shown. A too-low dynamic value leads to the identification of many insignificant local maxima (due to the presence of noise) outside the mask, even at START (A). Therefore, EMax dynamic should be set high enough to minimize false detections at START (B, C), while remaining low enough to avoid mistakenly excluding true objects at later time points (C). Detected objects overlaid on the original fluorescence image can be visually inspected.

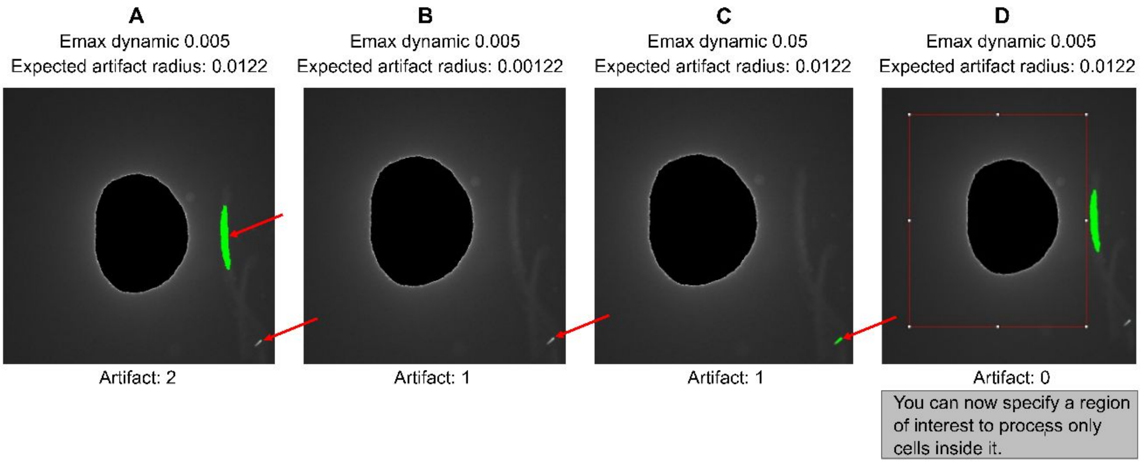

Figure S4

At START, two spurious objects were detected (A). One of these objects was excluded by lowering the "Expected Artifact Radius"(B) or by increasing the EMax dynamic value (C). Both objects can be eliminated from the analysis by drawing a region of interest (ROI) to define the area where calculations should be performed (D). (Note that in (A) and (B), the green detection marker of the bottom object may be only a single pixel, too small to visualize at this zoom level, and only indicated by the arrow.)

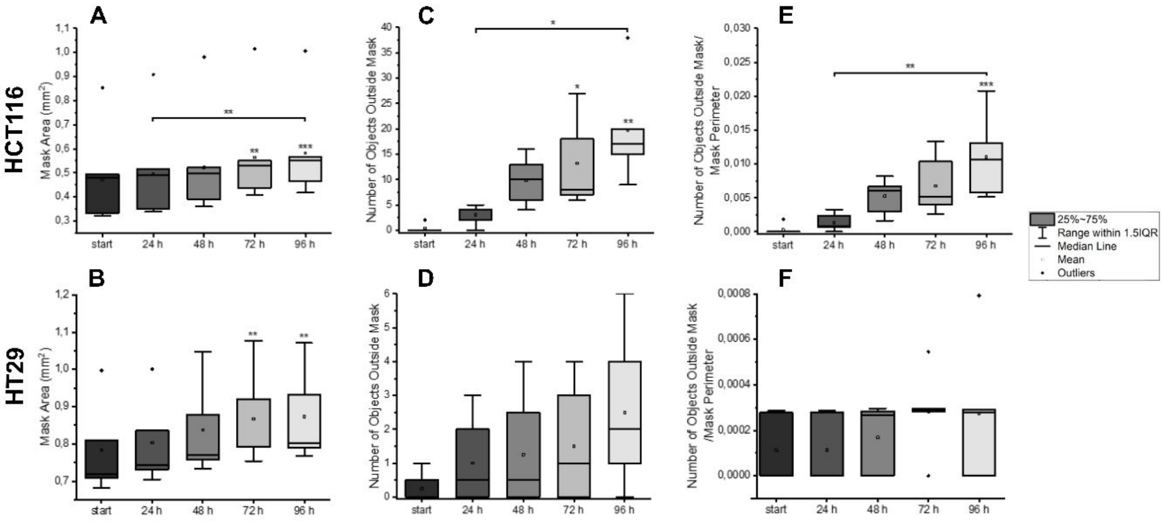

Figure S5

HCT116- and HT-29-derived spheroids were embedded in a collagen matrix. Their growth was assessed for four days based on spheroid area (A, B). Expansion into the extracellular matrix was quantified using the number of objects outside the mask, both in total and normalized values for HCT116 (C, E) and HT-29 (D, F). Significant differences between START and following time intervals as assessed by Friedman´s ANOVA are marked with asterisks, other significant comparisons are indicated by asterisk brackets; *$p < 0.05$, **$p < 0.01$, ***$p < 0.001$.
